# 2-Amino-3-carb­oxy­pyrazin-1-ium perchlorate bis­(2-amino­pyrazin-1-ium-3-carboxyl­ate) monohydrate

**DOI:** 10.1107/S1600536812021071

**Published:** 2012-05-12

**Authors:** Fadila Berrah, Sofiane Bouacida, Ahlem Bouhraoua, Thierry Roisnel

**Affiliations:** a Laboratoire de Chimie Appliquée et Technologie des Matériaux (LCATM), Université d’Oum El Bouaghi 04000, Algeria; b Unité de Recherche de Chimie de l’Environnement et Moléculaire Structurale (CHEMS), Faculté des Sciences Exactes, Université Mentouri Constantine 25000, Algeria; c Centre de Difractométrie X, UMR 6226 CNRS Unité Sciences Chimiques de Rennes, Université de Rennes I, 263 Avenue du Général Leclerc, 35042 Rennes, France

## Abstract

The asymmetric unit of the title compound, C_5_H_6_N_3_O_2_^+^·ClO_4_^−^·2C_5_H_5_N_3_O_2_·H_2_O, comprises two symmetry-independent zwitterions, one cation, one perchlorate anion and one water mol­ecule. In the crystal, the three different types of organic entities are linked by N—H⋯O and N—H⋯N hydrogen bonds, forming undulating sheets parallel to (1-10). These sheets are in turn connected by O—H⋯N and O—H⋯O hydrogen bonds involving perchlorate anions and water mol­ecules, forming a three-dimensional network. Intra­molecular N—H⋯O and weak inter­molecular C—H⋯O hydrogen bonds are also present.

## Related literature

For crystal structures of hybrid compounds obtained from 3-amino-pyrazine 2-carb­oxy­lic acid, see: Berrah *et al.* (2011*a* ▶,*b* ▶,*c* ▶). For related perchlorate compounds, see: Bendjeddou *et al.* (2003 ▶); Berrah *et al.* (2012 ▶); Toumi Akriche *et al.*(2010 ▶).
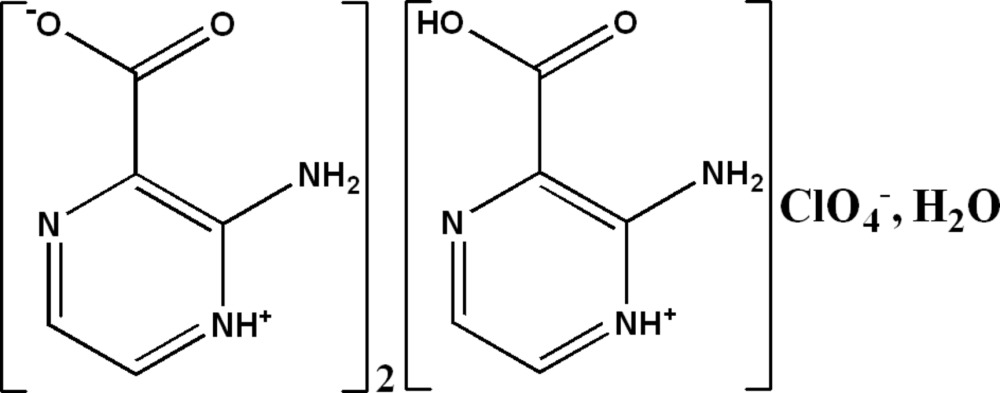


## Experimental

### 

#### Crystal data

C_5_H_6_N_3_O_2_^+^·ClO_4_^−^·2C_5_H_5_N_3_O_2_·H_2_O
*M* *_r_* = 535.83
Triclinic, 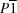

*a* = 8.1332 (14) Å
*b* = 11.816 (2) Å
*c* = 11.850 (2) Å
α = 95.696 (9)°
β = 108.148 (8)°
γ = 102.416 (8)°
*V* = 1039.8 (3) Å^3^
*Z* = 2
Mo *K*α radiation
μ = 0.27 mm^−1^
*T* = 150 K
0.46 × 0.27 × 0.17 mm

#### Data collection

Bruker APEXII diffractometer
Absorption correction: multi-scan (*SADABS*; Sheldrick, 2002 ▶) *T*_min_ = 0.855, *T*_max_ = 0.955
15575 measured reflections
4705 independent reflections
4165 reflections with *I* > 2σ(*I*)
*R*_int_ = 0.057

#### Refinement

*R*[*F* ^2^ > 2σ(*F* ^2^)] = 0.038
*wR*(*F* ^2^) = 0.1
*S* = 1.04
4705 reflections
332 parameters
H atoms treated by a mixture of independent and constrained refinement
Δρ_max_ = 0.39 e Å^−3^
Δρ_min_ = −0.48 e Å^−3^

### 

Data collection: *APEX2* (Bruker, 2006 ▶); cell refinement: *SAINT* (Bruker, 2006 ▶); data reduction: *SAINT*; program(s) used to solve structure: *SIR2002* (Burla *et al.*, 2005 ▶); program(s) used to refine structure: *SHELXL97* (Sheldrick, 2008 ▶); molecular graphics: *ORTEP-3 for Windows* (Farrugia, 1997 ▶) and *Mercury* (Macrae *et al.* 2006 ▶); software used to prepare material for publication: *WinGX* (Farrugia, 1999 ▶).

## Supplementary Material

Crystal structure: contains datablock(s) global, I. DOI: 10.1107/S1600536812021071/lh5470sup1.cif

Structure factors: contains datablock(s) I. DOI: 10.1107/S1600536812021071/lh5470Isup2.hkl

Supplementary material file. DOI: 10.1107/S1600536812021071/lh5470Isup3.cml

Additional supplementary materials:  crystallographic information; 3D view; checkCIF report

## Figures and Tables

**Table 1:** Hydrogen-bond geometry (Å, °)

*D*—H⋯*A*	*D*—H	H⋯*A*	*D*⋯*A*	*D*—H⋯*A*
O1*A*—H1*A*⋯O1*W*	0.82	1.71	2.5258 (18)	172
O1*W*—H1*W*⋯O1*B*^i^	0.88 (2)	1.92 (2)	2.7873 (19)	169 (2)
O1*W*—H2*W*⋯O2	0.84 (2)	1.99 (2)	2.8176 (19)	172 (2)
O1*W*—H1*W*⋯N3*B*^i^	0.88 (2)	2.56 (2)	3.052 (2)	115.8 (17)
N2*B*—H2*B*⋯O1*C*	0.86	1.92	2.6935 (18)	149
N1*C*—H11*C*⋯O2*B*^ii^	0.86	2.11	2.958 (2)	170
N2*C*—H2*C*⋯O1*B*^ii^	0.86	1.76	2.6156 (19)	171
N2*A*—H2*A*⋯O2*C*^iii^	0.86	1.80	2.6536 (17)	175
N1*A*—H11*A*⋯O1*C*^iii^	0.86	2.14	2.9340 (19)	153
N1*B*—H11*B*⋯O1*A*	0.86	2.26	2.916 (2)	133
N1*B*—H11*B*⋯O1*C*	0.86	2.44	3.087 (2)	133
N1*B*—H12*B*⋯O2*B*	0.86	2.21	2.814 (2)	127
N1*A*—H12*A*⋯O2*A*	0.86	2.09	2.7038 (19)	128
N1*C*—H12*C*⋯O2*C*	0.86	2.06	2.6734 (19)	128
N2*B*—H2*B*⋯N3*C*	0.86	2.41	3.058 (2)	132
N1*B*—H12*B*⋯N3*A*	0.86	2.41	3.035 (2)	130
N1*A*—H12*A*⋯N3*B*^iv^	0.86	2.44	3.152 (2)	140
C4*B*—H4*B*⋯O1^v^	0.93	2.41	3.267 (2)	153
C4*C*—H4*C*⋯O3^vi^	0.93	2.45	3.350 (2)	162
C5*A*—H5*A*⋯O2*B*	0.93	2.58	3.336 (2)	138
C5*B*—H5*B*⋯O2^v^	0.93	2.48	3.145 (2)	129
C5*B*—H5*B*⋯O2*A*^v^	0.93	2.48	3.164 (2)	130

Symmetry codes: (i) 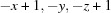; (ii) 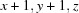; (iii) 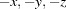; (iv) 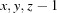; (v) 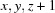; (vi) 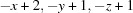.

**Fig. 1.**
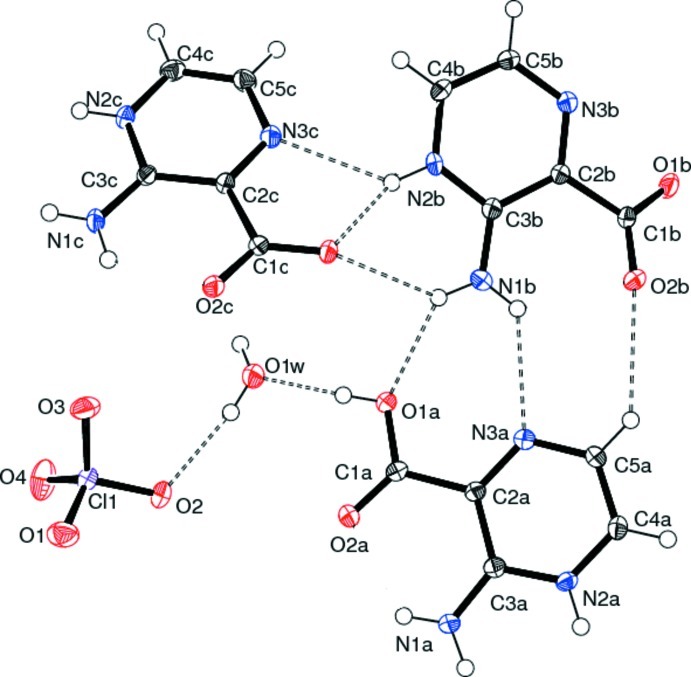
The asymmetric unit of the title compound. Displacement ellipsoids are drawn at the 50% probability level. Hydrogen bonds are shown as dashed lines.

**Fig. 2.**
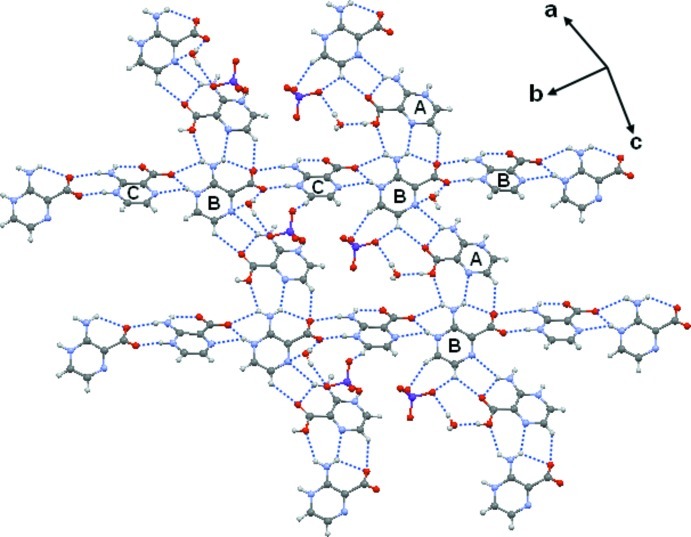
A partial view of one extended double-sheet showing wave-like chains and H-bonds between the different components. Hydrogen bonds are shown as dashed lines.

**Fig. 3.**
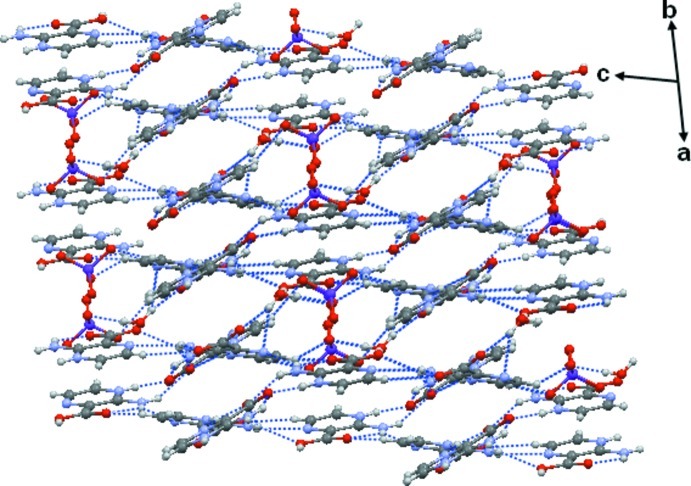
A view of part of the packing of (I) showing how double-sheets are linked via perchlorate anions and water molecules. Hydrogen bonds are shown as dashed lines.

## supplementary crystallographic information

### Comment

N-heterocyclic compounds such as pyrazine and its derivatives encompass a variety of potential hydrogen donors and acceptors which makes them interesting units to built new edifices involving original hydrogen-bonding schemes. As a continuation of our systematic studies concerning the synthesis and structural characterization of organic-inorganic hybrids and in attempts to establish a relationship between the nature of the anion used and hydrogen-bonding pattern encountered in these structures, we report herein the crystal structure of the compound 2-Amino-3-carboxypyrazin-1-ium perchlorate bis(2-aminopyrazin-1-ium 3-carboxylate) monohydrate. Related compounds obtained with nitrate, sulfate and dihydrogen phosphate anions have been reported (Berrah *et al.* 2011*a*,*b*,*c*).

The asymmetric unit, shown in Fig.1, comprises two symmetry independent zwitterions (B and C), one cation (A), one perchlorate anion and one water molecule. Bonds distances and angles in the three organic entities are comparable to that encountered in similar structures (Berrah *et al.* 2011*a*,*b*,*c*) except for the C—O distances in the carboxylic group: C—O distances are 1.2524 (19) and 1.2553 (19) Å in (B) and 1.2418 (19) and 1.2631 (19) Å in (C), due to the transfer of the carboxylic group proton to the hetero-ring nitrogen atom. Perchlorate anions present quite regular tetrahedral geometry (Cl—O distances range from 1.4279 (13) to 1.4528 (12) Å and angles from 108.67 (8) to 110.11 (9)°) and are comparable to that reported in the literature (Bendjeddou *et al.* 2003; Berrah *et al.*2012; Toumi Akriche *et al.*2010).

All components of the structure are involved in an interesting hydrogen bond system in which all potential donors and acceptors are implicated: the H_2_O molecule, the two 2-Aminopyrazin-1-ium 3-carboxylate zwitterions (B and C) and the 2-Amino-3-carboxypyrazin-1-ium cation (A) act as both hydrogen bond donors and acceptors (table 1). An extensive H-bonding system between the three different organic entities, allows development of wave-like extended chains which intersect to form double-sheets parallel to (110) (Fig.2 and Fig.3). Perchlorate anions and water molecules connect these double-sheets to generate a three-dimensional network (Fig.2 and Fig.3).

### Experimental

The title compound was obtained by reacting 3-amino-pyrazine 2-carboxylic acid with some excess of perchloric acid in aqueous solution. Slow evaporation allows growth of well formed colourless prismatic crystals.

### Refinement

All H atoms were located in differnce Fourier maps. The water molecule H atoms were refined with *U*_iso_(H) = 1.5*U*_eq_(O) while all the remaining H atoms were introduced in calculated positions and treated as riding on their parent atoms (C,*N* or O) with C—H = 0.93 Å,*N*—H = 0.86 Å and O—H = 0.82 Å with *U*_iso_(H) = 1.2 *U*_eq_(C or N) and *U*_iso_(H) = 1.5 *U*_eq_(O).

### Crystal data

**Table:**

C_5_H_6_N_3_O_2_^+^·ClO_4_^−^·2C_5_H_5_N_3_O_2_·H_2_O	*Z* = 2
*M**_r_* = 535.83	*F*(000) = 552
Triclinic, *P*1	*D*_x_ = 1.712 Mg m^−^^3^
Hall symbol: -P 1	Mo *K*α radiation, λ = 0.71073 Å
*a* = 8.1332 (14) Å	Cell parameters from 7298 reflections
*b* = 11.816 (2) Å	θ = 2.7–27.4°
*c* = 11.850 (2) Å	µ = 0.27 mm^−^^1^
α = 95.696 (9)°	*T* = 150 K
β = 108.148 (8)°	Prism, colourless
γ = 102.416 (8)°	0.46 × 0.27 × 0.17 mm
*V* = 1039.8 (3) Å^3^	

### Data collection

**Table:**

Bruker APEXII diffractometer	4165 reflections with *I* > 2σ(*I*)
Graphite monochromator	*R*_int_ = 0.057
CCD rotation images, thin slices scans	θ_max_ = 27.5°, θ_min_ = 2.7°
Absorption correction: multi-scan (*SADABS*; Sheldrick, 2002)	*h* = −10→10
*T*_min_ = 0.855, *T*_max_ = 0.955	*k* = −15→15
15575 measured reflections	*l* = −14→15
4705 independent reflections	

### Refinement

**Table:**

Refinement on *F*^2^	Primary atom site location: structure-invariant direct methods
Least-squares matrix: full	Secondary atom site location: difference Fourier map
*R*[*F*^2^ > 2σ(*F*^2^)] = 0.038	Hydrogen site location: inferred from neighbouring sites
*wR*(*F*^2^) = 0.1	H atoms treated by a mixture of independent and constrained refinement
*S* = 1.04	*w* = 1/[σ^2^(*F*_o_^2^) + (0.0379*P*)^2^ + 0.495*P*] where *P* = (*F*_o_^2^ + 2*F*_c_^2^)/3
4705 reflections	(Δ/σ)_max_ = 0.001
332 parameters	Δρ_max_ = 0.39 e Å^−^^3^
0 restraints	Δρ_min_ = −0.48 e Å^−^^3^

### Special details

**Table:**

Geometry. All e.s.d.'s (except the e.s.d. in the dihedral angle between two l.s. planes) are estimated using the full covariance matrix. The cell e.s.d.'s are taken into account individually in the estimation of e.s.d.'s in distances, angles and torsion angles; correlations between e.s.d.'s in cell parameters are only used when they are defined by crystal symmetry. An approximate (isotropic) treatment of cell e.s.d.'s is used for estimating e.s.d.'s involving l.s. planes.
Refinement. Refinement of *F*^2^ against ALL reflections. The weighted *R*-factor *wR* and goodness of fit *S* are based on *F*^2^, conventional *R*-factors *R* are based on *F*, with *F* set to zero for negative *F*^2^. The threshold expression of *F*^2^ > σ(*F*^2^) is used only for calculating *R*-factors(gt) *etc*. and is not relevant to the choice of reflections for refinement. *R*-factors based on *F*^2^ are statistically about twice as large as those based on *F*, and *R*- factors based on ALL data will be even larger.

### Fractional atomic coordinates and isotropic or equivalent isotropic displacement parameters (Å^2^)

**Table:**

	*x*	*y*	*z*	*U*_iso_*/*U*_eq_	
Cl1	0.69001 (5)	0.36663 (3)	−0.00929 (3)	0.01747 (11)	
O3	0.68759 (17)	0.43096 (11)	0.09976 (11)	0.0282 (3)	
C2A	0.07122 (19)	−0.13735 (13)	0.05029 (13)	0.0142 (3)	
O2	0.62112 (16)	0.24144 (10)	−0.01437 (11)	0.0248 (3)	
O1A	0.32377 (15)	0.00920 (10)	0.18281 (10)	0.0214 (3)	
H1A	0.4049	0.0661	0.1869	0.032*	
O1W	0.55592 (16)	0.19617 (10)	0.19895 (12)	0.0222 (3)	
H1W	0.663 (3)	0.2094 (19)	0.254 (2)	0.033*	
H2W	0.566 (3)	0.205 (2)	0.132 (2)	0.033*	
O2C	0.40678 (15)	0.39784 (9)	0.29630 (10)	0.0185 (2)	
N2C	0.88489 (17)	0.53957 (11)	0.58581 (12)	0.0170 (3)	
H2C	0.9651	0.6013	0.588	0.02*	
O1	0.57705 (18)	0.40133 (11)	−0.11220 (11)	0.0298 (3)	
N3C	0.63598 (17)	0.34284 (12)	0.58168 (12)	0.0176 (3)	
O1B	0.11825 (16)	−0.26089 (10)	0.61036 (11)	0.0235 (3)	
C5B	0.3196 (2)	0.08607 (14)	0.75159 (14)	0.0183 (3)	
H5B	0.3426	0.1123	0.833	0.022*	
C5C	0.7831 (2)	0.38892 (15)	0.68069 (15)	0.0197 (3)	
H5C	0.7998	0.3523	0.7478	0.024*	
O4	0.86893 (17)	0.38880 (12)	−0.01070 (14)	0.0359 (3)	
N1A	−0.01642 (18)	−0.16806 (12)	−0.17083 (12)	0.0212 (3)	
H11A	−0.0909	−0.2062	−0.2398	0.025*	
H12A	0.0748	−0.1132	−0.1671	0.025*	
N2B	0.34558 (17)	0.12346 (11)	0.56691 (12)	0.0176 (3)	
H2B	0.3852	0.1715	0.5257	0.021*	
C1C	0.4396 (2)	0.34074 (13)	0.38055 (14)	0.0156 (3)	
C4B	0.3817 (2)	0.16113 (14)	0.68530 (14)	0.0186 (3)	
H4B	0.4485	0.2378	0.7214	0.022*	
C2C	0.61173 (19)	0.39387 (13)	0.48635 (14)	0.0147 (3)	
C3C	0.7406 (2)	0.49782 (13)	0.48389 (14)	0.0150 (3)	
O2B	−0.00600 (16)	−0.22021 (10)	0.42749 (10)	0.0239 (3)	
O1C	0.34056 (15)	0.24560 (10)	0.38619 (10)	0.0219 (3)	
C3A	−0.04216 (19)	−0.19367 (13)	−0.07064 (14)	0.0150 (3)	
N2A	−0.18714 (16)	−0.28048 (11)	−0.08043 (11)	0.0152 (3)	
H2A	−0.2602	−0.3146	−0.1513	0.018*	
N1C	0.72791 (18)	0.55293 (12)	0.39109 (12)	0.0199 (3)	
H11C	0.8106	0.6143	0.3961	0.024*	
H12C	0.6369	0.5274	0.3257	0.024*	
C5A	−0.1068 (2)	−0.26064 (14)	0.12868 (14)	0.0178 (3)	
H5A	−0.1291	−0.2857	0.1957	0.021*	
N3B	0.22488 (17)	−0.02635 (12)	0.70059 (12)	0.0173 (3)	
C1B	0.0919 (2)	−0.19231 (14)	0.53518 (14)	0.0172 (3)	
O2A	0.25539 (16)	0.00695 (10)	−0.01483 (10)	0.0239 (3)	
C2B	0.1914 (2)	−0.06368 (13)	0.58521 (14)	0.0156 (3)	
N3A	0.03789 (17)	−0.17121 (11)	0.14528 (12)	0.0164 (3)	
C1A	0.2274 (2)	−0.03376 (13)	0.06933 (14)	0.0154 (3)	
C4C	0.9077 (2)	0.48821 (15)	0.68396 (15)	0.0200 (3)	
H4C	1.0073	0.52	0.7532	0.024*	
N1B	0.2181 (2)	−0.01866 (13)	0.39418 (13)	0.0263 (3)	
H11B	0.2591	0.0311	0.3549	0.032*	
H12B	0.1569	−0.0888	0.3577	0.032*	
C3B	0.2491 (2)	0.01272 (14)	0.51060 (14)	0.0170 (3)	
C4A	−0.2213 (2)	−0.31536 (13)	0.01601 (14)	0.0168 (3)	
H4A	−0.3216	−0.376	0.0063	0.02*	

### Atomic displacement parameters (Å^2^)

**Table:**

	*U*^11^	*U*^22^	*U*^33^	*U*^12^	*U*^13^	*U*^23^
Cl1	0.01646 (19)	0.01438 (19)	0.0198 (2)	0.00210 (14)	0.00545 (15)	0.00173 (14)
O3	0.0371 (7)	0.0237 (6)	0.0195 (6)	0.0060 (5)	0.0069 (5)	−0.0027 (5)
C2A	0.0137 (7)	0.0142 (7)	0.0136 (7)	0.0036 (6)	0.0033 (6)	0.0016 (6)
O2	0.0290 (6)	0.0143 (6)	0.0267 (7)	−0.0020 (5)	0.0092 (5)	0.0016 (5)
O1A	0.0193 (6)	0.0231 (6)	0.0145 (6)	−0.0055 (4)	0.0039 (4)	0.0011 (5)
O1W	0.0196 (6)	0.0226 (6)	0.0202 (6)	−0.0005 (5)	0.0047 (5)	0.0046 (5)
O2C	0.0207 (6)	0.0158 (5)	0.0141 (5)	0.0003 (4)	0.0019 (4)	0.0031 (4)
N2C	0.0142 (6)	0.0147 (6)	0.0187 (7)	−0.0012 (5)	0.0050 (5)	0.0012 (5)
O1	0.0366 (7)	0.0292 (7)	0.0208 (6)	0.0125 (6)	0.0033 (5)	0.0057 (5)
N3C	0.0171 (6)	0.0169 (7)	0.0171 (7)	0.0018 (5)	0.0052 (5)	0.0038 (5)
O1B	0.0256 (6)	0.0160 (6)	0.0206 (6)	−0.0040 (5)	0.0023 (5)	0.0050 (5)
C5B	0.0214 (8)	0.0167 (8)	0.0134 (7)	0.0013 (6)	0.0044 (6)	0.0004 (6)
C5C	0.0184 (8)	0.0233 (8)	0.0156 (8)	0.0037 (6)	0.0036 (6)	0.0062 (6)
O4	0.0208 (6)	0.0277 (7)	0.0630 (10)	0.0046 (5)	0.0204 (7)	0.0096 (7)
N1A	0.0205 (7)	0.0232 (7)	0.0126 (7)	−0.0041 (5)	0.0026 (5)	0.0016 (5)
N2B	0.0201 (7)	0.0144 (6)	0.0180 (7)	−0.0002 (5)	0.0084 (5)	0.0058 (5)
C1C	0.0167 (7)	0.0146 (7)	0.0137 (7)	0.0004 (6)	0.0057 (6)	0.0006 (6)
C4B	0.0206 (8)	0.0136 (7)	0.0171 (8)	−0.0005 (6)	0.0045 (6)	−0.0011 (6)
C2C	0.0151 (7)	0.0126 (7)	0.0154 (7)	0.0011 (6)	0.0058 (6)	0.0015 (6)
C3C	0.0156 (7)	0.0131 (7)	0.0155 (7)	0.0017 (6)	0.0063 (6)	0.0003 (6)
O2B	0.0263 (6)	0.0210 (6)	0.0156 (6)	−0.0051 (5)	0.0031 (5)	0.0006 (5)
O1C	0.0221 (6)	0.0186 (6)	0.0165 (6)	−0.0066 (4)	0.0029 (5)	0.0036 (4)
C3A	0.0148 (7)	0.0153 (7)	0.0147 (7)	0.0046 (6)	0.0043 (6)	0.0022 (6)
N2A	0.0139 (6)	0.0154 (6)	0.0127 (6)	0.0018 (5)	0.0016 (5)	0.0007 (5)
N1C	0.0192 (7)	0.0173 (7)	0.0176 (7)	−0.0042 (5)	0.0040 (5)	0.0051 (5)
C5A	0.0190 (7)	0.0193 (8)	0.0152 (8)	0.0019 (6)	0.0080 (6)	0.0035 (6)
N3B	0.0182 (6)	0.0166 (7)	0.0153 (7)	0.0011 (5)	0.0053 (5)	0.0034 (5)
C1B	0.0157 (7)	0.0164 (8)	0.0172 (8)	−0.0014 (6)	0.0070 (6)	0.0009 (6)
O2A	0.0270 (6)	0.0226 (6)	0.0164 (6)	−0.0036 (5)	0.0056 (5)	0.0055 (5)
C2B	0.0148 (7)	0.0155 (7)	0.0142 (7)	0.0002 (6)	0.0043 (6)	0.0020 (6)
N3A	0.0170 (6)	0.0171 (7)	0.0142 (6)	0.0032 (5)	0.0053 (5)	0.0022 (5)
C1A	0.0152 (7)	0.0150 (7)	0.0143 (7)	0.0035 (6)	0.0036 (6)	0.0003 (6)
C4C	0.0173 (7)	0.0226 (8)	0.0161 (8)	0.0035 (6)	0.0025 (6)	0.0002 (6)
N1B	0.0368 (8)	0.0218 (7)	0.0159 (7)	−0.0043 (6)	0.0116 (6)	0.0017 (6)
C3B	0.0166 (7)	0.0163 (8)	0.0157 (8)	0.0008 (6)	0.0048 (6)	0.0027 (6)
C4A	0.0150 (7)	0.0149 (7)	0.0202 (8)	0.0016 (6)	0.0072 (6)	0.0029 (6)

### Geometric parameters (Å, º)

**Table:**

Cl1—O4	1.4279 (13)	N2B—C4B	1.347 (2)
Cl1—O1	1.4362 (13)	N2B—C3B	1.354 (2)
Cl1—O3	1.4406 (13)	N2B—H2B	0.86
Cl1—O2	1.4528 (12)	C1C—O1C	1.2553 (19)
C2A—N3A	1.317 (2)	C1C—C2C	1.516 (2)
C2A—C3A	1.439 (2)	C4B—H4B	0.93
C2A—C1A	1.504 (2)	C2C—C3C	1.442 (2)
O1A—C1A	1.3103 (18)	C3C—N1C	1.321 (2)
O1A—H1A	0.82	O2B—C1B	1.2418 (19)
O1W—H1W	0.88 (2)	C3A—N2A	1.352 (2)
O1W—H2W	0.84 (2)	N2A—C4A	1.340 (2)
O2C—C1C	1.2524 (19)	N2A—H2A	0.86
N2C—C4C	1.345 (2)	N1C—H11C	0.86
N2C—C3C	1.353 (2)	N1C—H12C	0.86
N2C—H2C	0.86	C5A—N3A	1.349 (2)
N3C—C2C	1.314 (2)	C5A—C4A	1.365 (2)
N3C—C5C	1.350 (2)	C5A—H5A	0.93
O1B—C1B	1.2631 (19)	N3B—C2B	1.317 (2)
C5B—N3B	1.354 (2)	C1B—C2B	1.520 (2)
C5B—C4B	1.361 (2)	O2A—C1A	1.2112 (19)
C5B—H5B	0.93	C2B—C3B	1.432 (2)
C5C—C4C	1.364 (2)	C4C—H4C	0.93
C5C—H5C	0.93	N1B—C3B	1.321 (2)
N1A—C3A	1.321 (2)	N1B—H11B	0.86
N1A—H11A	0.86	N1B—H12B	0.86
N1A—H12A	0.86	C4A—H4A	0.93
			
O4—Cl1—O1	110.11 (9)	N1C—C3C—C2C	125.21 (14)
O4—Cl1—O3	110.07 (8)	N2C—C3C—C2C	115.84 (14)
O1—Cl1—O3	109.55 (8)	N1A—C3A—N2A	118.17 (14)
O4—Cl1—O2	109.26 (8)	N1A—C3A—C2A	125.89 (14)
O1—Cl1—O2	108.67 (8)	N2A—C3A—C2A	115.95 (13)
O3—Cl1—O2	109.15 (8)	C4A—N2A—C3A	122.52 (13)
N3A—C2A—C3A	121.68 (14)	C4A—N2A—H2A	118.7
N3A—C2A—C1A	118.80 (13)	C3A—N2A—H2A	118.7
C3A—C2A—C1A	119.47 (13)	C3C—N1C—H11C	120
C1A—O1A—H1A	109.5	C3C—N1C—H12C	120
H1W—O1W—H2W	110 (2)	H11C—N1C—H12C	120
C4C—N2C—C3C	122.50 (14)	N3A—C5A—C4A	121.69 (14)
C4C—N2C—H2C	118.8	N3A—C5A—H5A	119.2
C3C—N2C—H2C	118.8	C4A—C5A—H5A	119.2
C2C—N3C—C5C	119.78 (14)	C2B—N3B—C5B	119.75 (14)
N3B—C5B—C4B	120.86 (15)	O2B—C1B—O1B	126.42 (15)
N3B—C5B—H5B	119.6	O2B—C1B—C2B	118.62 (14)
C4B—C5B—H5B	119.6	O1B—C1B—C2B	114.96 (13)
N3C—C5C—C4C	121.16 (15)	N3B—C2B—C3B	121.61 (14)
N3C—C5C—H5C	119.4	N3B—C2B—C1B	117.00 (14)
C4C—C5C—H5C	119.4	C3B—C2B—C1B	121.38 (14)
C3A—N1A—H11A	120	C2A—N3A—C5A	119.10 (13)
C3A—N1A—H12A	120	O2A—C1A—O1A	124.13 (14)
H11A—N1A—H12A	120	O2A—C1A—C2A	121.45 (14)
C4B—N2B—C3B	122.34 (14)	O1A—C1A—C2A	114.38 (13)
C4B—N2B—H2B	118.8	N2C—C4C—C5C	119.23 (14)
C3B—N2B—H2B	118.8	N2C—C4C—H4C	120.4
O2C—C1C—O1C	125.77 (14)	C5C—C4C—H4C	120.4
O2C—C1C—C2C	116.62 (13)	C3B—N1B—H11B	120
O1C—C1C—C2C	117.59 (13)	C3B—N1B—H12B	120
N2B—C4B—C5B	119.43 (14)	H11B—N1B—H12B	120
N2B—C4B—H4B	120.3	N1B—C3B—N2B	119.36 (14)
C5B—C4B—H4B	120.3	N1B—C3B—C2B	124.66 (15)
N3C—C2C—C3C	121.48 (14)	N2B—C3B—C2B	115.96 (14)
N3C—C2C—C1C	116.98 (13)	N2A—C4A—C5A	119.01 (14)
C3C—C2C—C1C	121.52 (13)	N2A—C4A—H4A	120.5
N1C—C3C—N2C	118.95 (14)	C5A—C4A—H4A	120.5
			
C2C—N3C—C5C—C4C	0.2 (2)	C5B—N3B—C2B—C1B	177.24 (13)
C3B—N2B—C4B—C5B	−0.1 (2)	O2B—C1B—C2B—N3B	150.06 (15)
N3B—C5B—C4B—N2B	1.2 (2)	O1B—C1B—C2B—N3B	−29.6 (2)
C5C—N3C—C2C—C3C	0.9 (2)	O2B—C1B—C2B—C3B	−31.1 (2)
C5C—N3C—C2C—C1C	−177.26 (14)	O1B—C1B—C2B—C3B	149.24 (15)
O2C—C1C—C2C—N3C	171.42 (14)	C3A—C2A—N3A—C5A	−0.3 (2)
O1C—C1C—C2C—N3C	−6.8 (2)	C1A—C2A—N3A—C5A	177.01 (13)
O2C—C1C—C2C—C3C	−6.8 (2)	C4A—C5A—N3A—C2A	−1.3 (2)
O1C—C1C—C2C—C3C	175.03 (14)	N3A—C2A—C1A—O2A	−173.93 (14)
C4C—N2C—C3C—N1C	−179.80 (14)	C3A—C2A—C1A—O2A	3.5 (2)
C4C—N2C—C3C—C2C	−0.2 (2)	N3A—C2A—C1A—O1A	3.9 (2)
N3C—C2C—C3C—N1C	178.63 (15)	C3A—C2A—C1A—O1A	−178.67 (13)
C1C—C2C—C3C—N1C	−3.3 (2)	C3C—N2C—C4C—C5C	1.3 (2)
N3C—C2C—C3C—N2C	−0.9 (2)	N3C—C5C—C4C—N2C	−1.3 (2)
C1C—C2C—C3C—N2C	177.19 (13)	C4B—N2B—C3B—N1B	179.88 (15)
N3A—C2A—C3A—N1A	−177.91 (14)	C4B—N2B—C3B—C2B	−1.7 (2)
C1A—C2A—C3A—N1A	4.8 (2)	N3B—C2B—C3B—N1B	−179.13 (16)
N3A—C2A—C3A—N2A	2.0 (2)	C1B—C2B—C3B—N1B	2.1 (2)
C1A—C2A—C3A—N2A	−175.33 (12)	N3B—C2B—C3B—N2B	2.5 (2)
N1A—C3A—N2A—C4A	177.75 (14)	C1B—C2B—C3B—N2B	−176.22 (13)
C2A—C3A—N2A—C4A	−2.1 (2)	C3A—N2A—C4A—C5A	0.7 (2)
C4B—C5B—N3B—C2B	−0.3 (2)	N3A—C5A—C4A—N2A	1.1 (2)
C5B—N3B—C2B—C3B	−1.6 (2)		

### Hydrogen-bond geometry (Å, º)

**Table:**

*D*—H···*A*	*D*—H	H···*A*	*D*···*A*	*D*—H···*A*
O1*A*—H1*A*···O1*W*	0.82	1.71	2.5258 (18)	172
O1*W*—H1*W*···O1*B*^i^	0.88 (2)	1.92 (2)	2.7873 (19)	169 (2)
O1*W*—H2*W*···O2	0.84 (2)	1.99 (2)	2.8176 (19)	172 (2)
O1*W*—H1*W*···N3*B*^i^	0.88 (2)	2.56 (2)	3.052 (2)	115.8 (17)
N2*B*—H2*B*···O1*C*	0.86	1.92	2.6935 (18)	149
N1*C*—H11*C*···O2*B*^ii^	0.86	2.11	2.958 (2)	170
N2*C*—H2*C*···O1*B*^ii^	0.86	1.76	2.6156 (19)	171
N2*A*—H2*A*···O2*C*^iii^	0.86	1.80	2.6536 (17)	175
N1*A*—H11*A*···O1*C*^iii^	0.86	2.14	2.9340 (19)	153
N1*B*—H11*B*···O1*A*	0.86	2.26	2.916 (2)	133
N1*B*—H11*B*···O1*C*	0.86	2.44	3.087 (2)	133
N1*B*—H12*B*···O2*B*	0.86	2.21	2.814 (2)	127
N1*A*—H12*A*···O2*A*	0.86	2.09	2.7038 (19)	128
N1*C*—H12*C*···O2*C*	0.86	2.06	2.6734 (19)	128
N2*B*—H2*B*···N3*C*	0.86	2.41	3.058 (2)	132
N1*B*—H12*B*···N3*A*	0.86	2.41	3.035 (2)	130
N1*A*—H12*A*···N3*B*^iv^	0.86	2.44	3.152 (2)	140
C4*B*—H4*B*···O1^v^	0.93	2.41	3.267 (2)	153
C4*C*—H4*C*···O3^vi^	0.93	2.45	3.350 (2)	162
C5*A*—H5*A*···O2*B*	0.93	2.58	3.336 (2)	138
C5*B*—H5*B*···O2^v^	0.93	2.48	3.145 (2)	129
C5*B*—H5*B*···O2*A*^v^	0.93	2.48	3.164 (2)	130

Symmetry codes: (i) −*x*+1, −*y*, −*z*+1; (ii) *x*+1, *y*+1, *z*; (iii) −*x*, −*y*, −*z*; (iv) *x*, *y*, *z*−1; (v) *x*, *y*, *z*+1; (vi) −*x*+2, −*y*+1, −*z*+1.
